# 596. Immunogenicity and Safety of BPZE1, an Intranasal Live Attenuated Pertussis Vaccine, Evaluated With and Without Tdap in Healthy Children 6 to 17 Years Old: A Phase 2b, Randomized, Active-Controlled Study

**DOI:** 10.1093/ofid/ofae631.191

**Published:** 2025-01-29

**Authors:** Saul N Faust, Javier Céspedes, Lydiana Avila, Gabriela Ivankovich-Escoto, Helen Marshall, Peter Richmond, Terry Nolan, Jolanta Bernatoniene, Srini Bandi, Anil Shenoy, Lisa Weissfeld, Wei Lang, Camille Locht, Vivek Samal, Peter Goldstein, Ken Solovay, Keith Rubin, Stephanie Noviello

**Affiliations:** University of Southampton and University Hospital Southampton NHS Foundation Trust , Southampton, England, United Kingdom; Clinica San Agustin, San Jose, San Jose, Costa Rica; Instituto de Investigación de Ciencias Médicas, San Jose, San Jose, Costa Rica; Metropolitano Research Institute, San Jose, San Jose, Costa Rica; University of Adelaide, Adelaide, South Australia, Australia; University of Western Australia School of Medicine, Perth’s Children Hospital, Nedlands, Western Australia, Australia; The Peter Doherty Institute for Infection and Immunity, Murdoch Children’s Research Institute, Melbourne, Victoria, Australia; University Hospitals Bristol & Weston NHS Foundation Trust, Bristol Royal Hospital For Children, Bristol, England, United Kingdom; Leicester Children's Hospital, Leicester, England, United Kingdom; Bradford Royal Infirmary, Bradford, England, United Kingdom; WCG Clinical, Princeton, New Jersey; WCG Clinical, Princeton, New Jersey; Univ. Lille, CNRS, Inserm, CHU Lille, Institut Pasteur de Lille, U1019-UMR9017–CIIL - Centre for Infection and Immunity of Lille, Lille, Haute-Normandie, France; ILiAD Biotechnologies, Weston, Florida; ILiAD Biotechnologies, Weston, Florida; ILiAD Biotechnologies, Weston, Florida; ILiAD Biotechnologies, Weston, Florida; ILiAD Biotechnologies, Weston, Florida

## Abstract

**Background:**

BPZE1, a live attenuated intranasal (IN) pertussis vaccine, is designed to prevent *Bordetella pertussis* (*Bp*) infection, disease and transmission to address limitations of current pertussis vaccines. BPZE1 has been shown to protect adults, primed with whole cell pertussis vaccine in infancy, from colonization with virulent *Bp*. This study assessed BPZE1-induced immune responses, non-interference and safety when administered with and without tetanus-diphtheria-acellular pertussis vaccine (Tdap; Boostrix™) in children 6-17 years old, primed in infancy with acellular pertussis vaccine (aPV).

**Figure:**
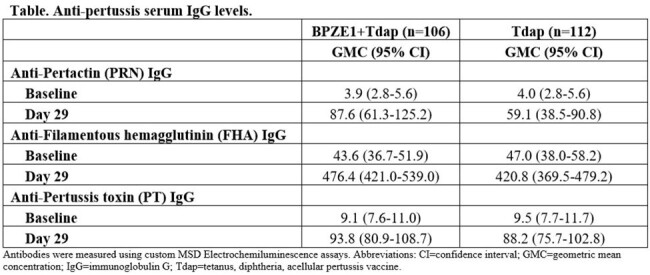

**Methods:**

366 healthy school-age participants were randomized 1:1:1 to 10^9^ CFU BPZE1+intramuscular placebo, 10^9^ CFU BPZE1+Tdap, or Tdap+IN placebo, and then vaccinated accordingly. Primary endpoints were geometric mean fold rise (GMFR) from baseline of nasal mucosal secretory immunoglobulin A (S-IgA) against whole cell pertussis extract (WCE) at Day 29 in BPZE1 and BPZE1+Tdap groups and solicited adverse events (AEs) through 7 days after vaccination. Key secondary endpoints were BPZE1+Tdap induction of serum IgG against tetanus, diphtheria, and aPV antigens compared with Tdap at Day 29.

**Results:**

GMFRs of S-IgA against WCE were similar among BPZE1 and BPZE1+Tdap groups (3.8 [95% CI 3.1-4.7] and 3.4 [2.8-4.2]), but low in Tdap group (1.2 [1.0-1.5]). All participants in BPZE1+Tdap and Tdap groups had anti-tetanus and anti-diphtheria antibody levels ≥0.1 IU/mL at Day 29. Serum IgG responses to PRN, FHA, and PT (aPV components) were similar between BPZE1+Tdap and Tdap groups (Table). Solicited AEs were 48%, 66%, 72% (local skin); 50%, 52%, 45% (nasal/respiratory); 42%, 50%, 55% (systemic) in BPZE1, BPZE1+Tdap and Tdap groups respectively. Three participants had SAEs unrelated to vaccination, no participants discontinued due to AE, and unsolicited AEs were similar between groups.

**Conclusion:**

Intranasal BPZE1 vaccination with or without Tdap demonstrated a favorable safety profile and induced robust nasal mucosal and non-interfering systemic immunogenicity in school-age children who had been primed with aPV as infants. In contrast to Tdap, BPZE1 has the potential to both protect against *Bp* colonization and reduce *Bp* transmission.

**Disclosures:**

**Saul N. Faust, FRCPCH PhD**, AstraZeneca: Grant/Research Support|BioNTech: Grant/Research Support|GSK: Grant/Research Support|Iliad Biotechnologies: Grant/Research Support|J&J: Grant/Research Support|J&J: Advisor, no personal payments (all honoraria paid to employing hospital)|Moderna: Grant/Research Support|Novavax: Advisor, no personal payments (all honoraria paid to employing hospital)|Pfizer: Advisor, no personal payments (all honoraria paid to employing hospital)|Sanofi: Grant/Research Support|Sanofi: Advisor, no personal payments (all honoraria paid to employing hospital)|Valneva: Grant/Research Support **Javier Céspedes, MD**, ILiAD Biotechnologies: Grant/Research Support **Lydiana Avila, MD**, ILiAD biotechnologies: Grant/Research Support **Gabriela Ivankovich-Escoto, MD**, Iliad: Honoraria **Helen Marshall, MD**, ILiAD biotechnologies: Grant/Research Support **Peter Richmond, MBBS**, ILiAD biotechnologies: Grant/Research Support **Terry Nolan, MD, PhD**, ILiAD biotechnologies: Grant/Research Support **Jolanta Bernatoniene, FRCPCH PhD**, Iliad Biotechnologies: Grant/Research Support **Srini Bandi, MD**, Iliad Biotechnologies: Grant/Research Support **Anil Shenoy, FRCPCH**, Iliad Biotechnologies: Grant/Research Support **Camille Locht, PhD**, ILiAD biotechnologies: Advisor/Consultant|ILiAD biotechnologies: Grant/Research Support **Peter Goldstein, n/a**, ILiAD Biotechnologies LLC: Employee|ILiAD Biotechnologies LLC: Stocks/Bonds (Private Company) **Ken Solovay, n/a**, ILiAD biotechnologies: Employee|ILiAD biotechnologies: Stocks/Bonds (Private Company) **Keith Rubin, MD**, ILiAD Biotechnologies: Board Member|ILiAD Biotechnologies: Ownership Interest|ILiAD Biotechnologies: Stocks/Bonds (Private Company) **Stephanie Noviello, MD, MPH**, ILiAD Biotechnologies: Employee|ILiAD Biotechnologies: Stocks/Bonds (Private Company)

